# Development of a Gene-Activated Scaffold Incorporating Multifunctional Cell-Penetrating Peptides for pSDF-1α Delivery for Enhanced Angiogenesis in Tissue Engineering Applications

**DOI:** 10.3390/ijms23031460

**Published:** 2022-01-27

**Authors:** Rachael N. Power, Brenton L. Cavanagh, James E. Dixon, Caroline M. Curtin, Fergal J. O’Brien

**Affiliations:** 1 Tissue Engineering Research Group, Royal College of Surgeons in Ireland (RCSI), D02 YN77 Dublin, Ireland; rachaelnpower@rcsi.ie (R.N.P.); carolinecurtin@rcsi.ie (C.M.C.); 2 Advanced Materials and Bioengineering Research Centre (AMBER), RCSI, D02 YN77 Dublin, Ireland; 3 Cellular and Molecular Imaging Core, RCSI, D02 YN77 Dublin, Ireland; brentoncavanagh@rcsi.ie; 4 School of Pharmacy, University of Nottingham Biodiscovery Institute, University of Nottingham, Nottingham NG7 2RD, UK; james.dixon@nottingham.ac.uk

**Keywords:** gene therapy, non-viral pDNA delivery, cell-penetrating peptide, gene-activated scaffold, angiogenesis in bone repair

## Abstract

Non-viral gene delivery has become a popular approach in tissue engineering, as it permits the transient delivery of a therapeutic gene, in order to stimulate tissue repair. However, the efficacy of non-viral delivery vectors remains an issue. Our lab has created gene-activated scaffolds by incorporating various non-viral delivery vectors, including the glycosaminoglycan-binding enhanced transduction (GET) peptide into collagen-based scaffolds with proven osteogenic potential. A modification to the GET peptide (FLR) by substitution of arginine residues with histidine (FLH) has been designed to enhance plasmid DNA (pDNA) delivery. In this study, we complexed pDNA with combinations of FLR and FLH peptides, termed GET* nanoparticles. We sought to enhance our gene-activated scaffold platform by incorporating GET* nanoparticles into collagen–nanohydroxyapatite scaffolds with proven osteogenic capacity. GET* N/P 8 was shown to be the most effective formulation for delivery to MSCs in 2D. Furthermore, GET* N/P 8 nanoparticles incorporated into collagen–nanohydroxyapatite (coll–nHA) scaffolds at a 1:1 ratio of collagen:nanohydroxyapatite was shown to be the optimal gene-activated scaffold. pDNA encoding stromal-derived factor 1α (pSDF-1α), an angiogenic chemokine which plays a role in BMP mediated differentiation of MSCs, was then delivered to MSCs using our optimised gene-activated scaffold platform, with the aim of significantly increasing angiogenesis as an important precursor to bone repair. The GET* N/P 8 coll–nHA scaffolds successfully delivered pSDF-1α to MSCs, resulting in a significant, sustained increase in SDF-1α protein production and an enhanced angiogenic effect, a key precursor in the early stages of bone repair.

## 1. Introduction

Bone is a highly vascularised tissue, capable of spontaneously regenerating itself following minor injuries through a combination of neovascularisation and osteogenesis [1]. However, critical-sized bone defects typically caused by trauma such as fractures, blast injuries, infection requiring extensive debridement, and tumour resection do not heal spontaneously [2]. The current clinical gold standard in critical-sized bone defects is autograft procedures, whereby bone tissue is grafted from the patient’s iliac crest to the defect site. However, this results in adverse side effects, such as donor site morbidity and infection [3]. Tissue engineering strategies aim to repair critical-sized bone defects by mimicking the natural regeneration process, combining biomaterials such as scaffolds, biochemical cues, and cells to repair damaged or injured tissue [4]. Scaffold biomaterials must provide a regenerative template, similar to native extracellular matrix, such that cells can infiltrate and generate new tissue growth [5]. Scaffold design approaches that are based on biodegradable polymers, such as collagen are often preferred, as the scaffold is resorbed by regenerating native tissue [6]. Calcium phosphate is widely used in scaffolds for bone repair, as it is naturally present in bone tissue, has high mechanical strength, and does not generate toxic by-products [7]. Strategies combining natural polymers and calcium phosphate were found to be the most successful for bone repair in vivo [8]. Previous research in our laboratory has focussed on the development of collagen–hydroxyapatite scaffolds [9,10,11] and collagen–nanohydroxyapatite (coll–nHA) scaffolds that have demonstrated significant bone repair in vivo [12,13]. The coll–nHA scaffolds will form the basis for this study, primarily used in conjunction with bone marrow-derived mesenchymal stromal cells (BM-MSCs).

Cells play a vital role in the tissue repair process by infiltrating the biomaterial scaffold, proliferating, and eventually building new extracellular matrix, while degrading the scaffold template [14,15]. BM-MSCs are self-replicating, non-haematopoietic stromal cells capable of differentiating into different mesenchymal lineages such as bone, cartilage, and adipose tissue [16]. The vital role of BM-MSCs in bone regeneration is well established in the literature [17,18,19], and as a result, BM-MSC therapeutics have been successfully applied in fracture repair and bone tissue engineering research [20,21,22,23]. In addition to their multilineage properties, BM-MSCs contribute to vascular formation and function, as they originate from a vascular location [24]. Although other stem cells such as embryonic stem cells (ESCs), induced pluripotent stem cells (iPSCs), and adipose-derived stem cells (ADSCs) have been used successfully in bone repair [25,26], BM-MSCs are perhaps more suitable in the scaffold-based approach as they are present in large quantities at the defect site and, therefore, the scaffold can be used as an off-the-shelf therapeutic in a minimally invasive manner.

Biochemical cues make up the final part of the tissue engineering triad, and aim to accelerate and enhance the regenerative process by stimulating cells infiltrating the scaffold to form new tissue, thus repairing the defect or injury [4,15]. Protein delivery has traditionally been utilised as a biochemical cue, for example, in Medtronic’s InFUSE™ device for the delivery of recombinant human bone morphogenetic protein 2 (rhBMP-2) protein in bone repair. However, protein delivery can have its drawbacks, such as ectopic bone formation resulting from supraphysiological dosage as well as high production costs [27]. Recently, gene delivery has become popular as an alternative approach, as it involves the introduction of genetic materials into host cells, thereby using the cells’ own internal machinery to upregulate or downregulate production of the protein of interest and bring about a therapeutic effect [28].The nucleic acids typically used in gene therapies include plasmid DNA (pDNA), messenger RNA (mRNA), microRNA (miRNA), small inhibitory RNA (siRNA), peptide nucleic acids (PNA) and locked nucleic acids (LNA) [29]. Gene delivery strategies can be subcategorised into viral and non-viral technologies, and both have lately been a topic of much interest, due to the highly effective use of mRNA vaccines to combat the spread of COVID-19 [30,31,32]. Viral gene delivery introduces a virus containing the gene of interest to host cells [33]. Non-viral gene delivery vectors utilise physical or chemical methods, sometimes involving nanoparticles to deliver therapeutic nucleic acids to host cells, meaning that the gene of interest is expressed extrachromosomally and transiently, facilitating greater control as non-viral delivery vectors can be engineered to facilitate short or long term expression [34]. In the instance of a defect or injury repair that tissue engineering seeks to address, non-viral gene delivery may be considered a more suitable therapeutic as it results in transient, short term expression of the gene of interest, as well as avoiding the challenges that can be associated with viral vector production, such as high biosafety implications and infrastructural requirements for manufacture [35]. However, non-viral gene delivery is known to be less effective and requires suprathreshold quantities of nucleic acid cargo to be delivered for significant and therapeutic levels of expression [36]. To achieve this, a non-viral delivery vector must be able to deliver while protecting cargo, escape endosomal entrapment, release cargo, and localise it to the correct organelle for activity, i.e., the nucleus for pDNA transfection, without adversely affecting cytotoxicity, even in the case of ‘difficult to transfect’ primary cells such as bone marrow-derived mesenchymal stem cells (BM-MSCs) [37]. The ability to transfect MSCs is particularly important in bone tissue engineering as BM-MSCs are thought to be the most important cell type involved in bone repair because of their ability to differentiate into osteoblast progenitors and secrete new extracellular matrix in the repair process.

**Figure 1:**
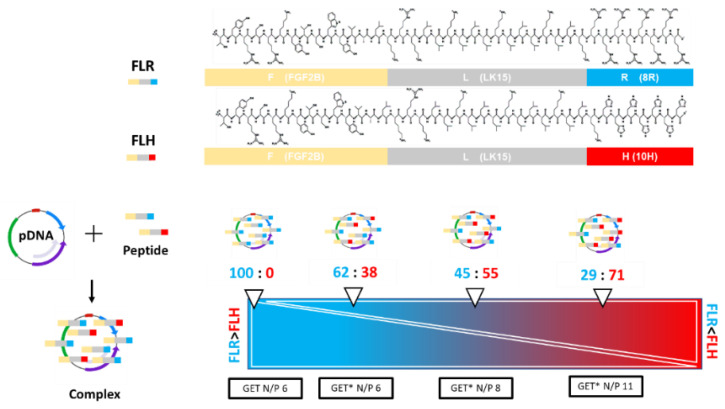
The GET* peptide is comprised of FLR and FLH combinations. Nanoparticles were complexed by mixing negatively charged pDNA with positively charged GET* peptide (FLR/FLH). The N/P ratio increases with increasing proportions of FLH in the FLR/FLH mixture.

Our lab has pioneered the use of gene-activated scaffolds, by incorporating non-viral gene delivery vectors including chitosan [38], polyethyleneimine (PEI) [39], hydroxyapatite [40,41], and the GET peptide [42] nanoparticles into collagen-based scaffolds, facilitating the effective delivery of therapeutic nucleic acids to cells as they infiltrate scaffolds. The gene-activated scaffold approach has proven effective for bone repair in vivo, significantly enhancing bone formation [38,43,44,45,46]. The GET peptide is capable of delivering a multitude of different cargoes, and consists of a 16-residue heparan sulphate-GAG binding peptide derived from the fibroblast growth factor 2 (FGF2) protein (FGF2B domain); LK15, an amphiphilic pan-nucleic acid interaction sequence; and8 arginine (8R) (named FLR) [47,48,49]. Dixon and colleagues have recently modified the GET peptide by replacing the 8R region of the cell-penetrating peptide (CPP) with a low charge 10H histidine sequence (named FLH). This sequence retains low charge until at low pH, triggering the imidazole ring of H to be protonated and the peptide to be charged. This is important for endosomal escape, whereby this system triggers endosomal swelling by osmotic imbalance across endosomal membranes, thus enhancing leakage of cargo from the entrapping vesicles. The FLR and FLH peptides were mixed together to form a vector we have termed modified GET (GET*), summarised in Figure 1. The formulations of GET* increase in positive charge concurrently with increasing proportions of FLH to FLR in the GET* vector.

In this study, we aim to formulate an enhanced gene-activated scaffold platform by optimising the GET* peptide for scaffold-based pDNA delivery. Previous research in our lab has focussed on the use of plasmids encoding for bone morphogenetic protein 2 (pBMP2) and vascular endothelial growth factor (VEGF) in gene-activated scaffolds for bone repair. This research highlighted the beneficial effects of increased vascularisation, as a result of pVEGF delivery alone or in combination with pBMP2, for bone regeneration [38,41]. Taking this into consideration, we adopted a gene-activated scaffold approach focussing on angiogenesis as a precursor to bone repair. The formation of fracture vasculature is the first step in the complex process of fracture healing, and is one of the most significant factors in successful bone regeneration [50,51,52]. As pVEGF has previously been studied extensively in this area, we sought to investigate the potential of an angiogenic plasmid encoding for chemokine stromal-derived factor 1α (pSDF-1α). This therapeutic has recently proven successful in gene-activated scaffolds for skin repair in our research group [53]. Interestingly, SDF-1α also plays a regulatory role in the BMP-2 induced osteoblastic differentiation of MSCs [54,55], which indicates strong potential for bone regeneration by enhancing the formation of early vasculature, as well as in subsequent stages of bone repair by the differentiation of MSCs into osteoblasts. Furthermore, the delivery of SDF-1α via gels and bone grafts has significantly enhanced bone repair in vivo [56,57].

Considering the potential that this therapeutic has shown and the importance of angiogenesis as a precursor to bone repair, this study sought to investigate the angiogenic potential of pSDF-1α on gene-activated scaffolds which incorporate GET* nanoparticles. The first aim was to formulate GET*-pDNA nanoparticles for delivery to MSCs in 2D. Secondly, the gene-activated scaffold platform was optimised by incorporating different formulations of GET* nanoparticles into collagen–nanohydroxyapatite scaffolds. This enabled identification of the most efficient gene-activated scaffold formulation for MSC transfection. Finally, pSDF-1α was delivered to MSCs using the optimal gene-activated scaffold, and efficacy was assessed by both SDF-1α protein production, and angiogenesis using a basement membrane assay.

## 2. Results

### 2.1. GET*-pDNA Nanoparticles Physico-Chemical Properties

**Figure 2:**
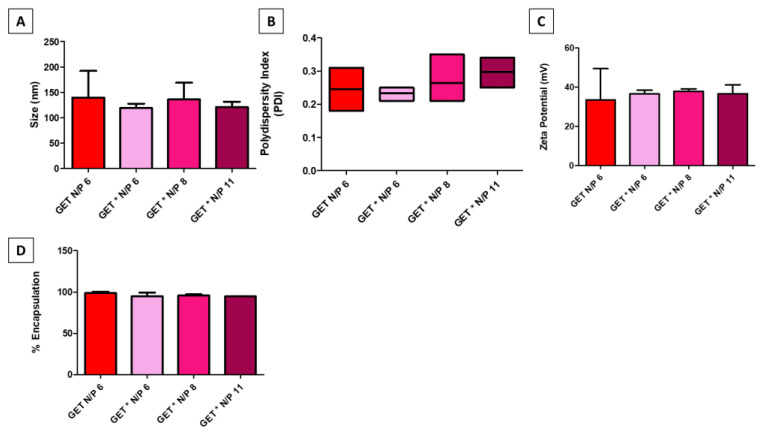
Physico-chemical characterisation demonstrated that there were no significant differences in (**A**) size (nm), (**B**) polydispersity index, (**C**) zeta potential (mV) and (**D**) % encapsulation efficiency between GET and modified GET* nanoparticles. Error bars represent ± SD, *n* = 3 samples tested in triplicate.

GET* was formulated by combining FLR and FLH, prior to complexation with pDNA to form nanoparticles. The different N/P ratios, where N/P ratio refers to the molar ratio of nitrogen residues in the vector to phosphate residues in the pDNA, were formulated by adjusting the proportion of FLH:FLR prior to complexation with pDNA. All GET* groups were compared with unmodified GET (FLR) alone. All GET* nanoparticles encapsulated pDNA to form nanoparticles with suitable physico-chemical properties for cellular uptake. Figure 2A–C showed the mean size (nm) of both unmodified GET N/P 6 and modified GET* N/P 6, GET** N/P 8, and GET*** N/P 11 was within the range 119 to 139.7 nm, the mean polydispersity index of the nanoparticle groups ranged from 0.23 to 0.29 and the mean zeta potential (mV) of all the nanoparticle groups was ≥25 mV. There were no statistically significant differences between groups. The QuantiFluor^®^ stain, employed as a sensitive method to detect and quantify uncomplexed pDNA following nanoparticle formulation, showed that both the unmodified GET and GET* nanoparticles encapsulated ≥95% of pDNA (Figure 2D) with no significant differences between groups.

### 2.2. 2D Comparison of GET and GET* Nanoparticles in MSCs

**Figure 3:**
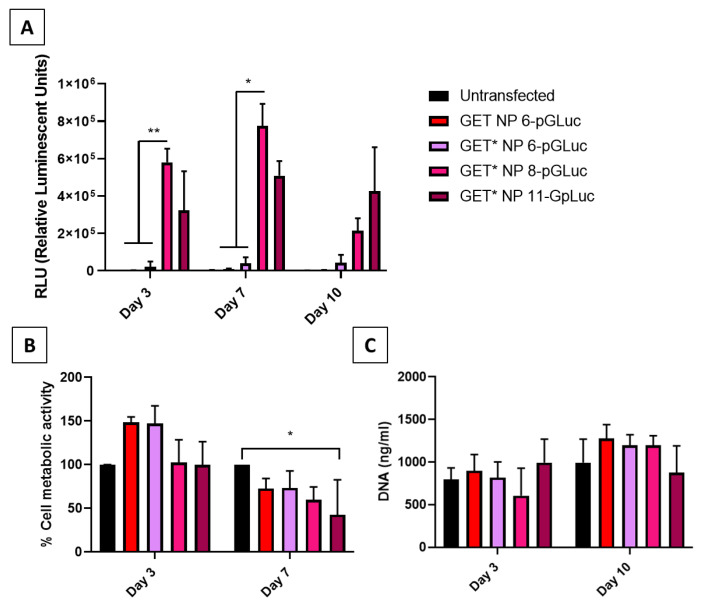
(**A**) The highest transfection efficiency was attained using GET* N/P 8 nanoparticles at both day 3 and day 7 post-transfection. (**B**) GET* nanoparticles did not have any adverse effect on cell metabolic activity (%) at day 3 but was significantly reduced in GET* N/P 11 transfected cells at day 7 post-transfection, compared with untransfected controls. (**C**) There were no significant differences in cell proliferation compared with untransfected controls, measured by DNA content at any timepoint. Error bars represent ±SD, *n* = 3 tested in triplicate, significance (*p* < 0.05 *, *p* < 0.01 **).

Transfections were carried out with reporter plasmids encoding for the *gaussia* luciferase enzyme (pGLuc) in MSCs in 2D. Relative luminescence (RLU), an indicator of the amount of luciferase enzyme produced by pGLuc-transfected cells, was measured at day 3, 7 and 10 post-transfection (Figure 3A). Transfection efficiency was significantly higher in cells transfected with GET* N/P 8 nanoparticles compared with other groups at day 3 and 7 post-transfection. Cytotoxicity of GET and GET* nanoparticles was assessed by measuring the change in cell metabolic activity (%), and proliferation (DNA content ng/mL) post-transfection compared with untransfected controls (Figure 3B,C). There were no significant differences in cell metabolic activity of GET*-transfected cells compared with untransfected cells at day 3 post-transfection. However, the metabolic activity of cells transfected with GET* N/P 11 nanoparticles was significantly lower (*p* < 0.05 *) than that of untransfected cells at day 7 (Figure 3B). There were no significant differences in cell proliferation (DNA ng/mL) between groups at either day 3 or day 10 (Figure 3C). Together, these results demonstrated that GET* N/P 8 nanoparticles were most effective for pDNA transfection, without adversely affecting cell viability.

### 2.3. Incorporation of GET* Nanoparticles into Coll–nHA Scaffolds and Assessment of Biocompatibility

**Figure 4:**
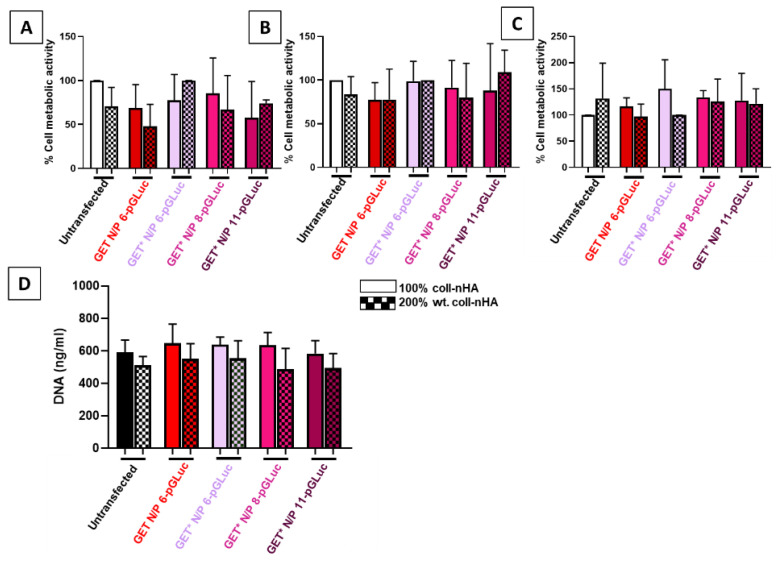
GET* nanoparticles on either 100% wt. or 200% wt. coll–nHA scaffolds had no adverse effect on cell metabolic activity at (**A**) day 3, (**B**) day 7, or (**C**) day 10. (**D**) Cell proliferation (DNA ng/mL) was not affected by transfection with gene-activated scaffolds. Error bars represent ±SD, *n* = 3 tested in triplicate.

Cytocompatibility of gene-activated scaffolds was assessed using cell metabolic activity and DNA content quantification (Figure 4A–C). The gene-activated scaffolds tested comprised of GET and GET* nanoparticles loaded onto 100% and 200% wt. collagen–nanohydroxyapatite (1:1 and 1:2 ratio of coll:nHA, respectively) scaffolds. Previous research conducted in our labs has shown that although the addition of nHA to collagen scaffolds significantly enhanced bone healing, bone formation in vivo did not increase with increasing nanohydroxyapatite concentration [12]. Therefore, both 100% and 200% wt. coll–nHA scaffolds were tested in order to determine which provided the optimal delivery platform, as their therapeutic efficacy is not significantly different. Cell metabolic activity and proliferation was not significantly affected by transfection with any of the gene-activated scaffolds tested at any timepoint (Figure 4A–D).

### 2.4. Measurement of GET* Coll–nHA Scaffold Transfection Efficiency

**Figure 5:**
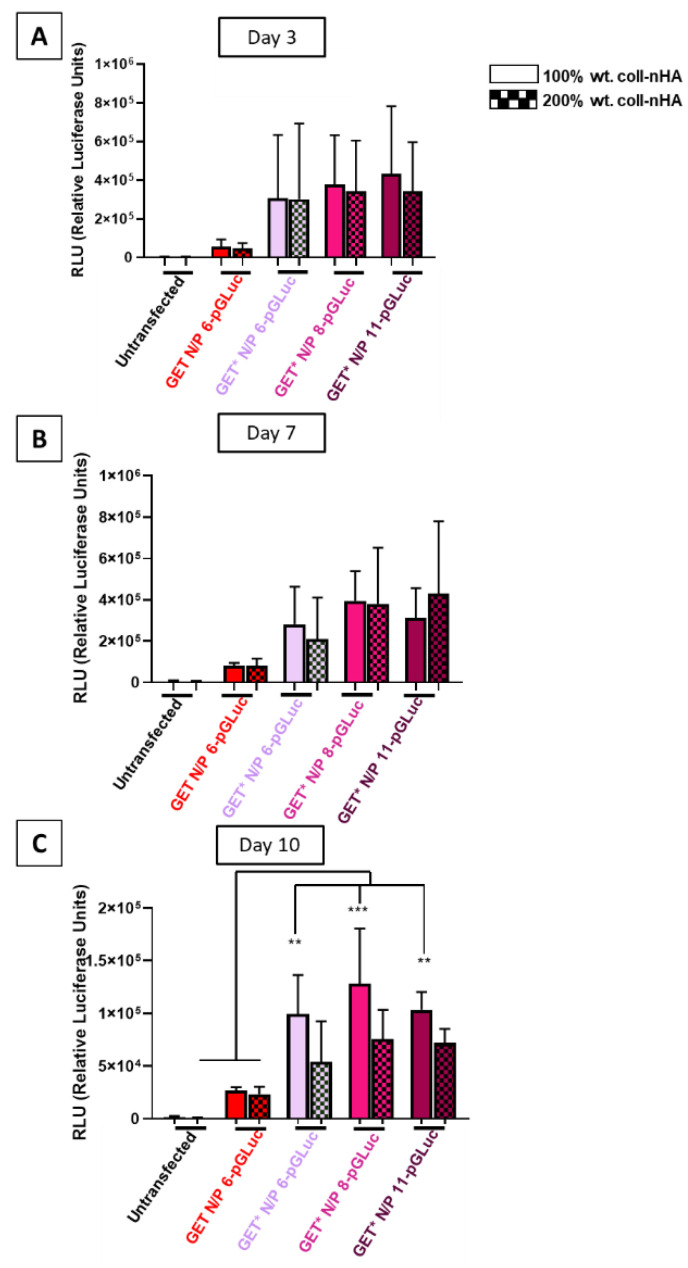
Transfection efficiency increased marginally in GET*-transfected cells, but not significantly at (**A**) day 3 or (**B**) day 7 post-transfection. (**C**) Transfection efficiency was significantly higher in GET* N/P 8-transfected MSCs on 100% wt. coll–nHA scaffolds at day 10 post-transfection (*p* < 0.01 **, *p* < 0.001 ***). Error bars represent ±SD, *n* = 3 tested in triplicate.

The transfection efficiency of the gene-activated scaffolds was quantified by measuring *Gaussia* luciferase levels by relative luminescence units (RLU) at day 3, day 7, and day 10 post-transfection (Figure 5A–C). Transfection efficiency was significantly higher in MSCs loaded on 100% wt. coll–nHA scaffolds containing GET* N/P 8 nanoparticles, compared with other groups. Although there appeared to be a general increase in transfection efficiency in all of the GET* gene-activated scaffolds at day 3 and day 7 (Figure 5A,B), transfection efficiency was not significantly increased. However, by day 10 (Figure 5C) transfection efficiency was significantly enhanced in all GET*-transfected cells seeded on 100% wt. coll–nHA scaffolds, compared with untransfected cells. However, the differences between GET* N/P 8 and untransfected cells was the most significant (*p* < 0.05 *, *p* < 0.01 **). Although there were no significant differences between 100% and 200% wt. coll–nHA groups, transfection efficiency was consistently higher in 100% wt. GET* N/P 8 gene-activated scaffolds. Hence, GET* N/P 8 scaffolds incorporated into 100% wt. coll–nHA scaffolds was shown to be our optimal gene-activated scaffold formulation.

### 2.5. Characterisation of the Stability and Angiogenic Potential of GET* N/P 8 Scaffolds

**Figure 6:**
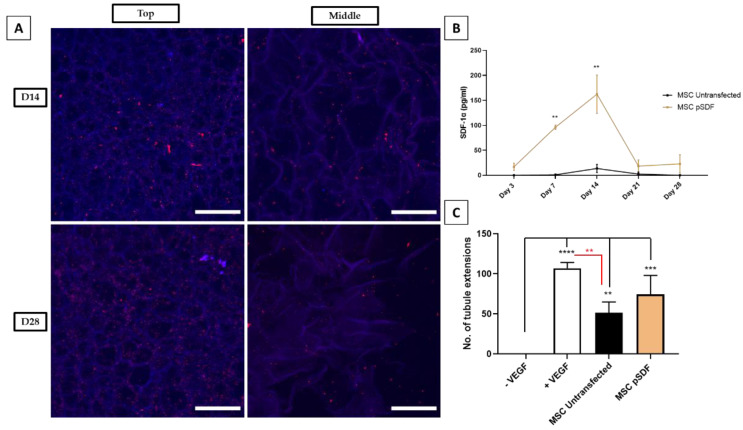
(**A**) Cy3-tagged GET* N/P 8-pDNA nanoparticles on coll–nHA (100% wt.) scaffolds were imaged using confocal microscopy both on the top, and in the middle of coll–nHA scaffolds, shown in blue, at day 14 and day 28 post-seeding. Scale bar = 100 µm. (**B**) SDF-1α (pg/mL) expression was significantly higher in transfected cells at day 7 and day 14 compared to untransfected control cells (*p* < 0.01 **). (**C**) Conditioned medium from pSDF-1α-transfected cells elicited formation of significantly more tubules than negative controls (*p* < 0.001 ***). The positive controls were significantly different to untransfected MSCs, but not pSDF-1α-transfected cells, shown in red (*p* < 0.01 **). (*p* < 0.0001 ****). Error bars represent ±SD, *n* = 3 tested in triplicate.

The stability of the GET* coll–nHA platform was assessed by imaging Cy3-tagged nanoparticles on the scaffolds for up to 28 days. Subsequently, MSCs were transfected with pSDF-1α using the GET* gene-activated scaffold platform to assess the therapeutic efficacy of the platform with respect to angiogenesis for bone repair, as discussed in the introduction. Figure 6 shows that Cy3-tagged nanoparticles remained associated with the scaffold struts (shown in blue) at the top surface, where they were seeded, and infiltrated the middle of the scaffolds by day 14 and day 28 post-seeding. There were slightly fewer nanoparticles visible within the scaffold at day 28, compared with day 14. Figure 6B shows the significantly increased expression of SDF-1α (pg/mL) following pSDF-1α transfection within the gene-activated scaffold, compared with untransfected cells at day 7 and day 14 post-transfection. SDF-1α expression subsequently decreased, returning to basal levels by day 28. These results indicated that although there are some nanoparticles still present on the scaffold at day 28 post-seeding, transfection decreased by that timepoint. The high number of nanoparticles visible on the scaffolds at day 14 correlated with high transfection efficiency at that timepoint.

The angiogenic effect of conditioned medium from pSDF-1α-transfected cells was assessed by quantifying new tubule extensions formed by rat endothelial progenitor cells (rEPCs) on a Cultrex^®^ basement membrane assay. The rEPCs were incubated in medium with and without VEGF to define positive and negative controls. Both the untransfected and pSDF-1α-transfected cells were shown to be significantly more angiogenic than negative controls, although the comparison between negative controls and pSDF-1α groups was more significant (*p* < 0.001 ***), represented by the black bars in Figure 6C. There was no significant difference between tubule extensions in the positive control and pSDF-1α groups, as depicted by the red bars in Figure 6C.

## 3. Discussion

The overall objective of this study was to formulate a scaffold-based delivery platform for pSDF-1α delivery, using modified GET* cell-penetrating peptides incorporated into collagen–nanohydroxyapatite scaffolds previously established for bone tissue engineering applications. Firstly, the use of novel modified GET* peptides was justified when compared with unmodified GET peptide. Physico-chemical characterisation showed that modified GET* nanoparticles had suitable properties for cellular uptake, in terms of size, charge and morphology. The optimal GET* nanoparticle formulation was identified as N/P 8, demonstrating the most effective transfection of rMSCs, both in 2D monolayer and on collagen–nanohydroxyapatite scaffolds. Coll–nHA 100% wt. scaffolds loaded with these GET* nanoparticles were found to be the optimal gene-activated scaffold formulation. This gene-activated scaffold platform was shown to stably retain nanoparticles for up to 28 days. Finally, this optimised platform was shown to be capable of effectively delivering therapeutic pSDF-1α, in a safe manner over a sustained period of time.

GET* nanoparticle formulations (N/P 6, 8, and 11) did not differ significantly from unmodified GET nanoparticles in terms of physico-chemical properties. As previously discussed, the GET* peptide is designed to attain a higher positive charge under lower pH conditions (~pH 5.5 in the cell endosome), whilst not having a higher pH than unmodified GET under normal pH. Hence, it is reasonable that the GET* nanoparticles did not have a significantly higher positive charge. Interestingly, GET* N/P 8 nanoparticles elicited the highest transfection efficiency in MSCs in 2D, significantly more so than unmodified GET and GET* N/P 6, and was also higher than the GET* N/P 11 group. This contrasts with similar optimisation studies in MSCs, where increasing N/P ratio tends to correlate with increased transfection efficiency [58,59]. One potential hypothesis to explain this result is that the increased positive charge conferred on GET* N/P 11 particles as a result of an increased proportion of FLH to FLR causes a more pronounced ‘proton-sponge’ effect and subsequently less viable cells can be transfected. However, there were no significant differences in zeta potential between GET* N/P 8 and 11, and increased positive charge tends to correlate with increased transfection efficiency [60]. Sergeeva et al. have described a similar result in primary cells using jetPEI^®^-pDNA complexes, where transfection efficiency was highest at N/P 5-10, and decreased at N/P 15-30 [61]. This could potentially be due to the higher toxicity, as discussed above, or increased packaging of the pDNA cargo rendering it inaccessible once delivered. With respect to the effect of GET* nanoparticles on cytotoxicity, cell metabolic activity was significantly lower in GET*N/P 11-pGLuc-transfected cells compared with controls at day 7 post-transfection. However, the modification of GET* peptides was designed to increase the nanoparticle’s capacity to escape the endosome and traffic pDNA to the cell nucleus, and previous reports in the literature have suggested that polymer swelling and subsequent endosomal membrane disruption may be the mechanism by which positively charged nanoparticles escape the endosome [62,63]. Therefore, a slight loss of cell viability or cell metabolic activity can be expected when using a nanoparticle designed to facilitate better endosomal escape. Cell proliferation was not significantly altered following transfection with GET* nanoparticles. Taken together, these results indicate that GET* N/P 8 nanoparticles were optimal in 2D, as transfection efficiency was significantly enhanced, without adversely effecting cell viability.

The improved transfection efficiency of GET* over unmodified GET in rMSCs supports the hypothesis that modification to enhance endosomal escape improves overall transfection efficiency. It is well established in the literature that endosomal escape is a frequent roadblock to effective pDNA transfection [62,64,65]. While different approaches are taken to overcome this, endosomal disruption (as is used here) is a popular option as it is applicable to many different cell types [66,67,68]. Our results concur with this, as they show that GET* is capable of effectively transfecting rMSCs, a ‘difficult-to-transfect’ primary cell type [37]. Furthermore, the modification in this case is comprised of amino acid substitutions that increase the peptide’s positive charge in the low pH environment of the cell endosome, thus achieving endosomolysis, or the “proton sponge effect” [63]. Other researchers using similar amino acid substitutions to the ones utilised here have reported similar effects [69,70], whereby enhanced CPPs were shown to exert their effects via endosomolysis [63,66,68,69,70].

GET* nanoparticles efficacy and cytotoxicity were then assessed when incorporated into collagen–nanohydroxyapatite scaffolds. Neither cell metabolic activity nor proliferation were affected by transfection on modified GET*pGLuc coll–nHA scaffolds. This result suggests a potential protective effect of the scaffold on cell viability, as GET* N/P 11 nanoparticles were shown to have a detrimental effect on cell viability in 2D, but not on coll–nHA scaffolds. Modified GET* N/P 8 nanoparticles were the most effective on both coll–nHA 100% and 200% wt. scaffold formulations. However, transfection was only significantly increased in the 100% wt. coll–nHA scaffolds. We hypothesise that the lower number of nHA nanoparticles present in this scaffold may potentially facilitate better loading of the GET* nanoparticles, and subsequently more cells taking up nanoparticles upon infiltrating the scaffolds. Previous studies carried out in our research group have shown that cell infiltration and osteogenic capacity in vivo does not differ significantly with increased nHA content [12]. Therefore, cell attachment between the two scaffold groups is similar, which is confirmed by the DNA content results in this study. As the number of cells and GET* nanoparticles present on the scaffold is the same, the effect must be due to the nHA content. Fewer nHA particles likely permits better incorporation and subsequent cellular uptake of GET* nanoparticles. As the main purpose of our study was ultimately to determine the optimal gene-activated scaffolds for bone tissue engineering applications, we can only speculate regarding this hypothesis. Future work could include further investigation of the release patterns, using UV spectroscopy or contact angle measurements [71], to help elucidate the reason.

The third aim of our study was to deliver angiogenic pSDF-1α to MSCs using our optimised gene-activated scaffolds, in order to enhance bone repair by first bringing about increased angiogenesis. As in vitro osteogenic studies typically last 28 days, we first demonstrated that GET*-activated coll–nHA scaffolds retained nanoparticles for this time period in standard cell culture conditions. Confocal images illustrated that while nanoparticles appeared to be well infiltrated and dispersed, there appeared to be fewer nanoparticles present at day 28, compared with day 14. This result was corroborated by the results of the SDF-1α ELISA, which demonstrated that SDF-1α production by transfected cells was significantly higher than untransfected controls at day 7 and day 14, returning to basal levels by day 28. Taken together, these results suggested that as cells infiltrate through the scaffold, they take up nanoparticles present within the porous microarchitecture at different levels. This confers a promising safety and efficacy profile of the optimal gene-activated scaffold, suggesting that the therapeutic genes being delivered will remain site-specific in vivo. Indeed, previous studies carried out within our research group have demonstrated the safety and efficacy of GET peptide delivery using collagen–hydroxyapatite scaffolds in vivo [43]. Finally, the delivery of pSDF-1α using the optimised gene-activated scaffold platform was also shown to bring about an enhanced angiogenic effect, whereby cells transfected with pSDF-1α gene-activated scaffolds were comparable with positive controls. Interestingly, levels of SDF-1α were already sufficiently increased at day 3 post-transfection to cause an angiogenic effect at early timepoints, as determined using the basement membrane assay, even before a significant increase in SDF-1α protein was detected by ELISA. These results were promising, as they suggest that the SDF-1α release brings about early angiogenesis, which is sustained at a high level over time, while still returning safely to basal levels by day 28. In the bone repair context, this is an important feature because the fracture vasculature that forms soon after injury provides vital nutrients and stem cells for the regeneration process [72,73].

Overall, these results demonstrated the successful formulation of a novel gene-activated scaffold platform capable of bringing about significantly enhanced angiogenesis. Firstly, the modified GET* peptide was successfully complexed with pDNA and demonstrated significantly enhanced efficacy over unmodified controls. Furthermore, the optimised platform of GET* N/P 8 nanoparticles on 100% wt. coll–nHA scaffolds successfully delivered angiogenic pSDF-1α cargo to MSCs, exhibiting a sustained increase in angiogenesis, crucially important in the early stages of bone regeneration. In future work, we aim to co-transfect MSCs with other bone marrow-derived cells to further increase angiogenesis, and investigate the effect of this on mineralisation in a chorioallantoic membrane (CAM) model. Additionally, this GET* coll–nHA platform can be further utilised to deliver different therapeutic cargoes in future work for various tissue engineering applications, as this study has demonstrated that the efficacy is reproducible using multiple plasmids and scaffold formulations.

## 4. Materials and Methods

### 4.1. Nanoparticle Formulation

#### 4.1.1. Plasmid Propagation

Plasmids encoding the following genes were propagated: *Gaussia* luciferase (pGLuc; New England Biolabs, MA, USA), and stromal-derived factor 1 alpha (SDF-1α:Cayla-InvivoGen, Toulouse, France) and both were under the control of the cytomegalovirus promoter. Plasmids were propagated by chemically transforming One Shot™ TOP10 chemically competent *E. coli* (ThermoFisher Scientific, Dublin, Ireland) bacterial cells according to the manufacturers protocol and expanded in lysogeny broth in the presence of antibiotics for which the plasmids carry resistance genes. The *E. coli* cells carrying pLuc were expanded in 100 µg/mL ampicillin (FisherScientific, Dublin, Ireland), and pSDF-1α *E. coli* cells were expanded in 100 µg/mL blasticidin (Sigma Aldrich, Wicklow, Ireland). pDNA was purified and collected using the Endotoxin free Maxi-prep kit (Qiagen, Manchester, UK). Plasmid was dissolved in molecular grade water at a concentration of 1 µg/mL and stored at −20 °C.

#### 4.1.2. GET and GET* Nanoparticle Formulation

The GET and GET* cell-penetrating peptides were synthesised using solid phase t-Boc chemistry and purified to >95% purity by Protein Peptide Research Ltd. (PPR, Hampshire, UK). GET nanoparticles were formulated at N/P ratio 6 (molar ratio of nitrogen in the amino acids to phosphate in the pDNA backbone) according to a previously published study [43]. GET* peptides were formulated by complexing FLR and FLH peptides at different ratios, prior to complexation with pDNA. Specifically; GET* N/P 6 consisted of 62% FLR: 38% FLH, GET* N/P 8 was 45% FLR: 55% FLH, and GET* N/P 11 was 29% FLR:71% FLH. Positive electrostatic charges increased concurrently with increasing FLH: FLR ratio, due to the increased amount of histidine relative to arginine. GET nanoparticles were fabricated as previously [43], by complexing 1mM FLR peptide with 1 µg/µL pDNA at room temperature for 15 min. GET* nanoparticles were fabricated by complexing GET* with pDNA. Briefly, the pDNA (1 µg/µL), 1 mM FLR and1 mM FLH peptides were diluted in OptiMEM™ (ThermoFisher Scientific, Dublin, Ireland) in separate Eppendorf^®^ tubes. FLR and FLH peptides were mixed briefly by pipetting, before being added to the pDNA and complexed at room temperature for 15 min. The amounts of pDNA and vector correspond to the pDNA dosage of 1 µg pDNA per 2.1 × 10^4^ rat mesenchymal stem cells (rMSCs) in a 12 well culture plate, or 2 µg pDNA per scaffold, as previously optimised in our laboratory [72]. Nanoparticles were complexed in molecular grade water (MGH_2_O, Sigma Aldrich, Wicklow, Ireland) for physico-chemical characterisations, or in OptiMEM reduced serum medium for cell transfection. Transfection was carried out in full serum medium following nanoparticle complexation.

### 4.2. Physico-Chemical Characterisation

#### 4.2.1. Size, Polydispersity Index and Zeta Potential

Size, polydispersity index, and zeta potential were measured using a Malvern Instruments Zetasizer™. Size measurements were taken using dynamic light scattering, which measures the hydrodynamic diameter of particles in solution and represents the average particle size as a “Z-average” mean. Polydispersity index describes the distribution of particles with the same size, according to a cumulants fit analysis. Zeta potential is measured using electrophoretic light scattering, whereby a current is applied across electrodes in the cuvette containing the sample, and the surface charge of the particles is measured. GET and modified GET* peptides were complexed with pDNA to formulate nanoparticles as described above. Nanoparticle formulations were made up to 1 mL in an Eppendorf tube, using MGH_2_O (Sigma Aldrich, Wicklow, Ireland). Glass cuvettes (PCS1115) were used for size and polydispersity measurements, and folded disposable capillary cells (DTS 1070) were used for zeta potential measurements. Cuvettes were primed by cleaning with 1 mL of 70% ethanol, followed by 5 rinses with MGH_2_O. Nanoparticle solutions (1 mL) were added using 1 mL syringes to the respective cuvettes, and size and zeta potential measurements were taken using a Malvern Zetasizer™, according to the user manual [73]. Size measurements were taken first, followed by zeta potential.

#### 4.2.2. pDNA Encapsulation Efficiency

Encapsulation efficiency refers to the ability of a vector to encapsulate pDNA, thereby forming a nanoparticle that protects the pDNA cargo from nucleases in the surrounding extracellular environment. pDNA encapsulation efficiency was measured by complexing reporter pGLuc with the GET and GET* nanoparticles as described in Section 4.1.2. The Promega QuantiFluor^®^ dsDNA System (MyBio Ltd., Kilkenny, Ireland) was then added to the nanoparticle solution as per the manufacturers protocol, prior to complexation for 5 min. A solution containing pDNA alone was complexed with the stain as a positive control. A solution containing 1X TE buffer and QuantiFluor^®^ stain alone was the negative control for this assay. Following complexation with the stain, 100 µL of each control and sample were pipetted into a 96-well black polystyrene plate and fluorescence was measured on a Tecan Infinite^®^ 200 Pro plate reader at an excitation wavelength of 504 nm and an emission wavelength of 531 nm. The percentage encapsulation efficiency was calculated by normalising the fluorescence in the uncomplexed pDNA sample to 100% exposure, and subtracting the percentage exposure for each sample from 100%.

### 4.3. Mesenchymal Stem Cell Isolation and Culture

Rat mesenchymal stem and endothelial progenitor cells were isolated from 6–8 week old male Sprague Dawley rats, as approved by the Research Ethics Committee of the Royal College of Surgeons in Ireland under application number TH017, according to protocols described in [46]. The femurs of both hindlimbs were clipped at both ends in order to expose the bone marrow. The bone marrow was flushed out with fully supplemented rat MSC medium (Dulbecco’s Modified Eagle Medium (DMEM) containing 20% foetal bovine serum (FBS), 2% penicillin streptomycin, 1% non-essential amino acids, 1% Glutamax, and 0.002% Primocin) using an 18 G needle and a 12 mL syringe into a 65 mm Petri dish. The isolate was cultured in 15 mL of media under standard cell culture conditions (37 °C, 5%CO_2_) for 24 h. The supernatant was then removed and centrifuged at 300× *g* for 5 min. The resultant cell pellet was cultured in Endogro basal medium supplemented with 0.002% Primocin on 1% porcine gelatine to select for endothelial progenitor cells. All cells were tested for mycoplasma at P1 and tested for differentiation at P4. MSCs were transfected between P4 and P6.

### 4.4. Optimisation of GET*-pDNA Delivery in 2D Monolayer

#### 4.4.1. rMSC Transfection with pGLuc

rMSCs were transfected at a seeding density of 2.1 × 10^4^ cells per well in 12 well cell culture plates, 24 h after seeding as per the seeding densities described in previous work carried out in the lab [46]. rMSCs were transfected with pGLuc, or pSDF-1α using the protocol described in Section 4.1.2. In the case of pGLuc transfection, transfection efficiency was measured from levels of *Gaussia* luciferase in cell supernatants using the Pierce™ *Gaussia* Luciferase Flash Assay (BioSciences, Dublin, Ireland) kit as per the manufacturer’s instructions. Briefly, 20 µL samples were incubated with 100 µL of coelenterazine substrate diluted 1:100 in duplicate, in a 96-well black flat bottomed assay plate. Luminescence was read immediately following substrate addition using a Tecan Infinite^®^ 200 Pro plate reader.

#### 4.4.2. Cytocompatibility of GET*-pDNA Nanoparticles

Cell metabolic activity was measured using an alamarBlue assay (Biosciences, Dublin, Ireland) at day 1, 3 and 7 post-transfection. The assay was carried out as per the manufacturer’s instructions, where cells were incubated with 10% alamarBlue for one hour before fluorescent measurements were taken from duplicated samples in a 96-well black flat bottomed assay plate, at an excitation wavelength of 545 nm and emission wavelength of 590 nm. Results were normalised using media alone incubated with alamarBlue. Cell proliferation following transfection was assessed by measuring cell number using the Invitrogen Quant-iT PicoGreen dsDNA kit (BioSciences, Dublin, Ireland) according to the manufacturer’s protocol. Cell number was compared to untransfected controls 10 days after transfection. Briefly, 100 μL of 1X PicoGreen reagent solution was added to samples lysed in 0.2 M carbonate and 1% Triton-X100 (BioSciences, Dublin, Ireland) cell lysate buffer in a 96-well black flat bottomed assay plate. Fluorescence was read at 538 nm using a Tecan Infinite^®^ 200 Pro plate reader. The final DNA (ng/mL) concentration was calculated from the standard curve generated using standards formulated according to the manufacturer’s instructions.

### 4.5. Scaffold-Based Optimsation of GET* Nanoparticle Delivery

#### 4.5.1. Collagen–Nanohydroxyapatite Scaffold Formulation

Collagen-nHA scaffolds were manufactured according to the technique developed in our lab at 100% wt. coll–nHA or 200% wt. coll–nHA [12]. Briefly, a 0.5% (*w*/*v*) collagen slurry containing type 1 bovine collagen in 0.05M glacial acetic acid was prepared by homogenisation at 15,000 rpm for 4 h. Two different concentrations of nHA were added relative to the weight of collagen used. The resulting precipitate was washed and resuspended using 2 min of sonication. Nanohydroxyapatite particles were added to the slurry during blending. The resulting coll–nHA slurries underwent a series of previously developed freeze drying cycles at −40 °C in 10 mm cylindrical stainless steel molds. Scaffolds were crosslinked and sterilised using a dehydrotheramal treatment (0.05 bar at 105 °C for 24 h). Scaffolds were hydrated in PBS (Sigma Aldrich, Wicklow, Ireland) before being chemically cross-linked with a solution of 1-(3-Dimethylaminopropyl)-3-ethylcarbodiimide hydrochloride (EDAC, Sigma-Aldrich, Wicklow, Ireland) and N-hydroxysuccinimide (NHS, Sigma-Aldrich, Wicklow, Ireland) in the ratio 5:2 for 2 h. Scaffolds were then washed twice in PBS to eliminate by-products of the reaction.

#### 4.5.2. Scaffold-Based Transfection of rMSCs

GET N/P 6 and GET* N/P 6, 8, and 11 nanoparticles were complexed as described in Section 4.1.2 before soak loading onto 100% and 200% wt. coll–nHA scaffolds. Nanoparticles were formulated to deliver a 2 µg pDNA dose per scaffold, as optimised in previous work from our lab [46]. Firstly, scaffolds were placed in a 24 well suspension culture plate and excess PBS was aspirated away. The nanoparticle solution for each side of the scaffold was prepared separately, in order to adhere to the complexation time of 15 min. Briefly, GET and GET* particles containing 1 µg pDNA were fabricated and soak loaded by pipetting onto the scaffold in a circular motion to ensure particles were uniformly dispersed throughout the scaffold. rMSCs were trypsinised between P4 and P6, before 2.5 × 10^5^ cells were added to each side of the scaffold immediately after the nanoparticles and incubated for 40 min. Gene-activated scaffolds were then turned over and the process repeated, prior to the addition of 2 mL growth medium. In total, 2 µg of pDNA was incorporated per scaffold, prior to seeding of 5 × 10^5^ rMSCs per scaffold, as per previous protocols established in our research group [46]. Scaffolds were transferred to fresh 24 well suspension plates containing fresh growth medium after 24 h to prevent cells attaching to the plate instead of the scaffold.

#### 4.5.3. Cytocompatibility of GET* Gene-Activated Scaffolds

Cytocompatibility of the GET and GET* gene-activated scaffolds was assessed by measuring cell metabolic activity and proliferation using Invitrogen alamarBlue^®^ and Quanti-iT PicoGreen™ assays (BioSciences, Dublin, Ireland), as described in Section 4.4.2 AlamarBlue^®^ assays were carried out at day 3, 7 and 10 post-transfection, PicoGreen™ assays were carried out on lysed scaffolds at 10 days post-transfection.

### 4.6. Assessment of Gene-Activated Scaffold Stability and Therapeutic Efficacy

#### 4.6.1. Confocal Imaging of Cy3-Tagged GET* N/P 8 Nanoparticles on Scaffolds

Following optimisation of our gene-activated scaffold platform, we tested the stability and therapeutic efficacy of this gene-activated scaffold over 28 days, to ascertain suitability for future in vitro and in vivo studies. In order to determine stability, pDNA was labelled red using the Mirus LabelIT^®^ Cy3 nucleic acid labelling kit (Medical Supply Company, Dublin, Ireland). Briefly, pDNA resuspended at 1 µg/µL was incubated at 37 °C for 1 h in the presence of the Cy3 reagent, buffered by Buffer A from the kit and MGH_2_O (Sigma Aldrich, Wicklow, Ireland), before being centrifuged through the G50 microspin columns provided. GET* N/P 8 nanoparticles were complexed using Cy3 pDNA, seeded on 100% wt. coll–nHA scaffolds as per Section 4.5.2, and cultured in OptiMEM^®^ for 28 days, with media changes every 3–4 days. At day 14 and day 28, scaffolds were fixed in 10% formalin for 1 h, before being rinsed twice in PBS and stored at 4 °C protected from light. Prior to imaging, scaffolds were sectioned in the middle such that Z stack images were taken of the top or bottom of the scaffold, where the nanoparticles were seeded, and from the middle. Images were acquired on a Carl Zeiss LSM 710, equipped with a W Plan-Apochromat 20× (NA 1.0) with a inter slice Z spacing of 1.2 µm to yielding a total image Z depth of 31.2 µm. The scaffold autofluorescence was excited using a 405 nm laser (detection range 410–509 nm). Cy3 fluorescence was excited using a 561 nm laser (detection range 564–681 nm). Images were recorded at a resolution of 1024 × 1024 pixels with a dwell time of 0.79 µs. Z stack images were maximum intensity projected and prepared in FIJI [74].

#### 4.6.2. Measurement of SDF-1α Expression and Angiogenesis

The therapeutic efficacy of our optimised gene-activated scaffold platform was assessed by delivering plasmids encoding for stromal-derived factor 1 alpha (pSDF-1α), before measuring the effects on protein expression and angiogenesis. Nanoparticles and cells were loaded onto the scaffolds as described in Section 4.5.2, and subsequently cultured for 28 days. Cell culture medium was collected at day 3, 7, 14, 21, and 28 post-transfection, for enzyme-linked immunosorbent assays (ELISA) analysis. Medium was exchanged every 3 days. Protein concentration (pg/mL) of SDF-1α was analysed from media samples collected using Research and Development System duoset ELISA kits (BioTechne, Abingdon, UK) as per the manufacturer’s instructions.

The angiogenic effect of pSDF-1α delivery using our optimised platform was measured by incubating rat endothelial progenitor cells (rEPCs) with conditioned medium samples taken at day 3 post-transfection. rEPCs were isolated from the same rats described in Section 4.3, and cultured in Endogro™ basal medium (Merck Millipore, Wicklow, Ireland) on 1% gelatine (Sigma Aldrich, Wicklow, Ireland), under standard cell culture conditions. At passage 4, the EPCs were validated by quantifying their ability to form tubule-like structures on a Matrigel^®^ basement membrane in the presence of vascular endothelial growth factor (VEGF). Angiogenesis assays were carried out using the Cultrex^®^ basement membrane by thawing the gel at 4 °C overnight, prior to plating in a 48 well cell culture plate at 120 µL per well. The gel was incubated at 37 °C for 30 min to ensure it had set, before seeding EPCs at 3 × 10^4^ cells per well. Cells were seeded in Endogro™ medium with no supplements as a negative control (-EGF), and with all supplements (+VEGF) representing the positive control. Cells for the experimental group were seeded in conditioned medium mixed with Endogro™ at a ratio of 3:1, to ensure that cell attachment occurred, as conditioned medium from transfected samples is MSC medium.

**Figure 7:**
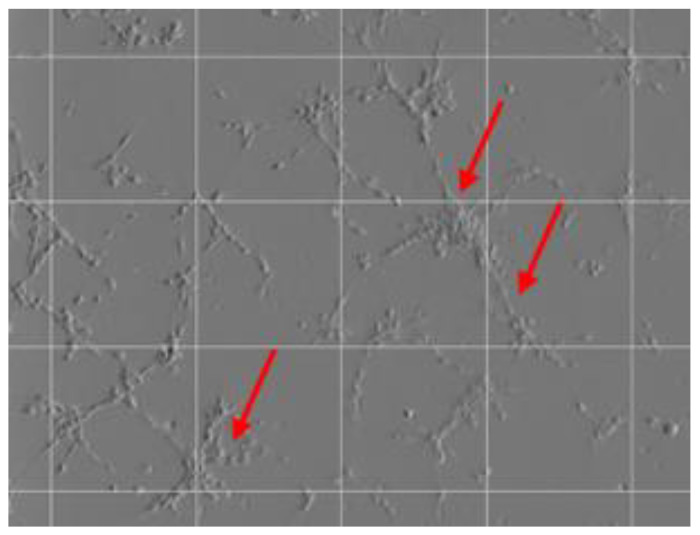
A sample phase contrast image of the EPCs forming microtubules on Cultrex^®^ basement membrane 10 h after cell seeding in conditioned medium. The gridlines were used to quantify the formation of new tubules without duplicating samples. Representative image chosen.

Tubule formation was subsequently measured by imaging on a Celldiscoverer 7 microscope (Carl Zeiss Ltd., Cambridge, UK) equipped with a 5×/0.35 numerical aperture. Plan-Apochromat objective and 1x optovar was used to record phase gradient contrast images with an Axiocam 506 camera. Z stack images composed of 20 slices were used to capture the depth in the sample over a range of 874 µm for each position. Each position was imaged hourly for 24 h. All Z stack images were processed using Extended Depth of Focus and the wavelet method (default settings) to yield a single image in Zen blue ed. software version 2.6. Images were taken in duplicate for each well, and conditions were measured in triplicate. Quantification of tubule formation was performed by a blinded operator using FIJI software [75]. Briefly, the phase contract image was opened using FIJI software, the ‘grid’ function was selected from the tools menu and a 1.1 × 10^5^ µm^2^ area per point centred grid (default settings) was placed over the image before counting the number of tubules formed. An example is shown in Figure 7, with sample tubules of interest highlighted.

### 4.7. Statistical Analysis

Results were analysed using GraphPad Prism^®^ software version 8 (San Diego, CA, USA) by carrying out a one-way ANOVA followed by Tukey’s post hoc test. All assays were carried out as *n* = 3, in triplicate and error bars are expressed as ±SD.

## 5. Conclusions

In summary, extensive characterisation revealed that GET* N/P 8 nanoparticles incorporated into collagen–nanohydroxyapatite scaffolds were the optimal gene-activated scaffold platforms for MSCs, indicating the potential for bone repair indications. This platform was proven to be stable and highly effective, bringing about increased, sustained angiogenesis following the delivery of pSDF-1α. This indicates significant promise for bone repair indications, where early angiogenesis is instrumental to the bone regeneration process. Additionally, the successful delivery of pDNA using this gene-activated scaffold platform indicates further potential for a broader range of tissue engineering applications, such as cartilage, skin, or nerve, by utilising different scaffold compositions.

## Data Availability

Data supporting reported results are stored in the Royal College of Surgeons OneDrive system.
